# Leadership for Early Career Psychiatrists – A rough guide on theory, practice and what to avoid

**DOI:** 10.1192/j.eurpsy.2023.206

**Published:** 2023-07-19

**Authors:** N. Sartorius

**Affiliations:** Association for the Improvement of Mental Health Programmes (AMH), Geneva, Switzerland

## Abstract

**Abstract:**

The presentation will focus on elements of leadership that have been shown to be useful on local and more comprehensive levels. It will be based on experience gained in leadership positions in international and national organizations and during leadership and professional skills courses which were conducted in more than 40 countries over the past thirty years. Among the topics that will be addressed for discussion and further action will be the need to introduce leadership training in the course of postgraduate education and to provide career advice at the beginning and during early years of service.

**Disclosure of Interest:**

None Declared

